# Temporal trends and risks factors for antimicrobial resistant *Enterobacteriaceae* urinary isolates from outpatients in Guadeloupe

**DOI:** 10.1186/s12866-016-0749-9

**Published:** 2016-06-24

**Authors:** Stéphanie Guyomard-Rabenirina, Joyce Malespine, Célia Ducat, Syndia Sadikalay, Mélanie Falord, Dorothée Harrois, Vincent Richard, Charles Dozois, The Laboratory working group, Clément Bourgoin, Farid Saheb, Guy-Joseph Theodore, Emmanuelle Bourgoin, Frédéric Leroy, William Laurent, Arnaud Lethuillier, Patricia Tamby, Petra Kassab, Anne-Christine Becker, Pierre Marie, Sandrine Hippomène, Olivier Menuteau, Marie-Line Péan, Sylvaine Bastian, Edith Malpote, Didier Mattera, Sébastien Breurec, Antoine Talarmin

**Affiliations:** Unité Environnement Santé, Institut Pasteur de Guadeloupe, Les Abymes, Guadeloupe France; Laboratoire de Biologie Clinique, Centre Hospitalier de Basse-Terre, Basse-Terre, Guadeloupe France; Faculté de Médecine, Université des Antilles, Pointe-à-Pitre, Guadeloupe France; Laboratoire de Microbiologie Clinique et Environnementale, Centre Hospitalo-Universitaire de Pointe-à-Pitre/Abymes, Pointe-à-Pitre, Guadeloupe France; Institut Pasteur de Nouvelle-Calédonie, Nouméa, Nouvelle-Calédonie France; Institut Armand Frappier, Laval, Canada

**Keywords:** Urinary tract infections, *Enterobacteriaceae*, Antibiotic resistance

## Abstract

**Background:**

Urinary tract infections are bacterial infections most commonly encountered in the community. The resistance rate of uropathogens to commonly prescribed antibiotics has increased worldwide but there are no published data concerning the resistance of strains isolated from community-acquired UTI in Guadeloupe. To assess the susceptibility patterns of *Enterobacteriaceae* strains isolated from outpatients in Guadeloupe we conducted a prospective study from December 2012 to May 2014 among outpatients consulting at private and public laboratories for urine analysis. Risk factors for *E. coli* resistance to amoxicillin, third-generation cephalosporin, and ciprofloxacin were also determined. To study the trends of *E. coli* resistance rates over the past 10 years, data on the susceptibility patterns of *E. coli* from 2003 to 2014 were also collected from three major laboratories for a retrospective study.

**Results:**

During the prospective study, we isolated 1293 bacterial strains from the urine of outpatients presenting for urine analysis. The most commonly isolated bacteria were *E. coli* (57 %) and *Klebsiella pneumoniae* (15.5 %). Thirty seven per cent of the *E. coli* strains were resistant to amoxicillin. Resistance rates to third generation cephalosporin were low for *E. coli* and other *Enterobacteriaceae* (3.1 and 12.2 % respectively) and mostly due to the presence of an Extended Spectrum Beta-lactamase. Resistance to cotrimoxazole and ciprofloxacin was moderate (17.8 and 15.6 % respectively). However, the resistance rate of *E. coli* to ciprofloxacin has significantly increased during the last 10 years. Risk factors were consistent with previously reported data, especially for the increasing ciprofloxacin resistance with age.

**Conclusion:**

General practitioners in Guadeloupe need to be better informed to favor the prescription of fosfomycin-trometamol to reduce the risk of resistance to fluoroquinolones.

## Background

Urinary tract infections (UTI) are bacterial infections most commonly encountered in the community, regardless of age. UTIs are usually benign and the infection (cystitis, prostatitis, urethritis) is limited to the lower urinary tract requires a simple antibiotic treatment. However, recurrences are common and may evolve into an upper UTI (pyelonephritis) requiring a heavier antibiotic treatment and more extensive management [1].

*Enterobacteriaceae*, especially *Escherichia coli*, are the most prevalent uropathogens in patients with UTI, both in the community and hospitals [2]. In most cases, the treatment of uncomplicated UTIs is empirical. The French Language Infectious Disease Society (SPFIL) recommends using a single dose of fosfomycin-trometamol in combination for the first line treatment of UTI and pivmecillinam for second line therapy. Beta-lactams, quinolones or cotrimoxazole should be administered after susceptibility testing since strains resistant to these antibiotics are frequently isolated. Indeed, multi drug resistant strains are emerging, especially strains harboring Extended Spectrum Betalactamases (ESBL). ESBL enzymes can hydrolyze almost all beta-lactams (except for carbapenems and cephamycins) and their genes are often closely linked to other genes that confer resistance to several other classes of antibiotics. During the past decade, CTX-M enzymes have gradually replaced the classical TEM and SHV-type ESBLs in many countries. The rapid international spread of CTX-M-15 has been associated with the global dissemination of particular *E. coli* clones, such as sequence type 131 [3].

The resistance rate of uropathogens to commonly prescribed antibiotics has increased worldwide: resistance to quinolones and trimethoprim increased in Europe between the ECO-SENS I (1999–2000) and ECO-SENS II (2007–2008) studies and in Africa (Senegal, Central African Republic) between 2003 and 2006, reducing therapeutic options [4–7].

Guadeloupe, a French overseas territory located in the Caribbean, is a very high resource country according to the Human Development Index in 2013. There are no published data concerning the resistance of strains isolated from community-onset UTI in Guadeloupe and data from the Caribbean more generally are rare. We, therefore, conducted a prospective study, from December 2012 to May 2014, to assess the susceptibility patterns of *Enterobacteriaceae* strains isolated from outpatients and to determine the risk factors for UTIs due to *Enterobacteriaceae* resistant to commonly used antimicrobial agents. We also studied the genetic basis for the antibiotic resistance of ESBL-producing *Enterobacteriaceae*. Finally, to determine the recent trends of resistance to antibiotics, we conducted a retrospective study on the antibiotic resistance of *E. coli* isolated from outpatients during the last 10 years.

## Methods

### Patients

We collected 1293 bacterial strains isolated from the urine of outpatients presenting for a urine analysis from 13 of the 23 private Guadeloupian laboratories and the laboratory of the Hospital of Basse-Terre between December 2012 and May 2014. All strains responsible for lower and upper UTI and bacteriuria according to SPFIL criteria (pyuria ≥10^4^ leucocyte/mL and bacteriuria ≥10^3^ for *E. coli* or *S. saprophyticus* and ≥10^4^ for other species) [8] were included in the study. Only one specimen was collected from each patient. The leucocyte count, isolation, identification and antibiotic susceptibility testing of bacteria were conducted by all laboratories according to their routine diagnostic procedures. All *Enterobacteriaceae* strains were sent to the Pasteur Institute of Guadeloupe for complete antibiotic susceptibility testing.

The study protocols were approved by the French Advisory Committee on Information Processing in Material Research in the Field of Health (CCTIRS 12–220). Written informed consent to participate in the study was obtained from all patients.

### Antibiotic susceptibility testing of Enterobacteriaceae at the Pasteur Institute of Guadeloupe

We assessed the susceptibility to the following antibiotics by the disk diffusion technique on Mueller-Hinton agar (MH) as recommended by the Antibiogram Committee of the French Microbiology Society (ACFMS) [9]: amoxicillin (10 μg), amoxicillin-clavulanic acid (20 μg/10 μg), ticarcillin (75 μg), cephalothin (30 μg), cefotaxime (30 μg), ceftazidime (30 μg), cefoxitin (30 μg), aztreonam (30 μg), imipenem (10 μg) gentamicin (15 μg), amikacin (30 μg), trimethoprim/sulfamethoxazole (1,25/23,75 μg), nalidixic acid (30 μg), ciprofloxacin (5 μg), fosfomycin (50 μg), nitrofurantoin (300 μg) and tetracycline (30 UI).. Inhibition growth diameters were measured using the Adagio automated system (Bio-Rad, France). Intermediate and resistant strains were grouped together and classified as resistant strains. Extended spectrum beta-lactamases were detected for strains with reduced inhibition growth diameters around cefotaxime, ceftazidime or aztreonam by the combined disk method with the following combinations: cefotaxime (30 μg) and ceftazidime (30 μg); cefotaxime (30 μg) and clavulanate (10 μg); and ceftazidime (30 μg) and clavulanate (10 μg).

The minimum inhibitory concentration (MIC) for cefotaxime and ceftazidime were determined using the agar-dilution method as recommended by the ACFMS for ESBL-carrying strains [9]. The cut-off values used for classification were: susceptible strains were defined as those having a MIC <8 mg/L for cefotaxime, and a MIC ≤4 mg/L for ceftazidime; and resistant strains as those having a MIC ≥32 mg/L for cefotaxime and a MIC ≥8 mg/L for ceftazidime. *E. coli* ATCC 25922 was used as a control stain.

### Molecular characterization of ESBL-producing strains

DNA was extracted using the Nucleospin tissue kit (Macherey Nagel, Hoerdt, France). Previously described polymerase chain reaction (PCR) methods were used to screen for plasmid-encoded *bla*_*CTX-M,*_*bla*_tem_ and *bla*_SHV_ genes [10–12]. Both *bla*_CTX-M,_*bla*_SHV_ and *bla*_TEM_ were then characterized by Direct DNA sequencing of the PCR products.

The nucleotide and deduced amino acid sequences were analyzed and compared with sequences available through the Internet on the National Center for Bio-technology Information web site (http://www.ncbi.nlm.nih.gov).

### Epidemiological data

A standardized specific questionnaire was completed to collect demographic data. Risk factors for *E. coli* resistance to amoxicillin, third-generation cephalosporin (3GC) and ciprofloxacin were assessed from questions about: age, gender, chronic illness, hospitalization during the last year, UTI during the previous last 3 months, urinary catheterization during the previous year, antibiotic exposure during the last month, and hospital or family hospital occupation.

### Evolution of antibiotic resistance in E. coli from 2003

To analyze the trends of resistance to antibiotics during the last 10 years, data were collected on the resistance of *E. coli* strains isolated from community-acquired UTIs in outpatients presenting at the emergency units of the hospitals of Basse-Terre and Pointe à Pitre and at the Pasteur Institute of Guadeloupe between 2003 and 2014. These data were added to that collected during the prospective study for 2013 and 2014.

### Data analysis

Statistical analysis was performed using R software (reference: R Development Core Team. R: A Language and Environment for Statistical Computing [Internet]. Vienna, Austria: R Foundation for Statistical Computing; 2010. Available: http://www.R-project.org/).

We used logistic regression to estimate the relation between resistance to each antibiotic and the various covariates (age, gender, clinical symptoms). All covariates with a p value lower than 0.20 were included in the multivariable model. Multivariable, backward, step-by-step binomial negative regressions were used to take into account confounders, bias, and interactions linked to the dependent variable.

To compare annual resistance rates, we performed Poisson regression and chi2 tests. Statistical differences were considered significant for *p*-values <0.05.

## Results

### Patients

We collected a total of 1293 strains during the study period. Most (73.5 %) were isolated from women (sex ratio 0.34). The median age of patients was 57.4 years (range 0 to 95 years). Sixteen patients carried significant numbers of two different strains. We obtained epidemiological data for 340 patients; pregnant women accounted for 10.6 % of women interviewed, and 69 % of patients had UTI symptoms.

### Bacterial strains and antibiotic susceptibility

More than 86 % of the isolates were *Enterobacteriaceae*: 736 *E. coli* (57.0 %), 202 *Klebsiella pneumoniae* (15.5 %), 56 *Proteus mirabilis* (4.3 %), 54 *Enterobacter* spp. (4.2 %), 38 *Citrobacter koseri* (2.9 %), and 35 other *Enterobacteriaceae* (2.7 %). Other Gram-negative bacteria (*Pseudomonas* spp. and *Acinetobacter* spp.) accounted for 0.6 % of the isolates. Among the isolates, 170 were Gram-positive bacteria (13.1 %): *Streptococcus agalactiae* (4.3 %), *Enterococcus faecalis* (4.2 %), and *Staphylococcus saprophyticus* (2.3 %).

Many of the *E. coli* isolates were susceptible to numerous drugs, but 42.1 % of the strains were resistant to amoxicillin and ticarcillin.

A small proportion of strains were not susceptible to 3GC (3.1 % *E. coli* and 12.2 % other *Enterobacteriaceae*; Table 1). Most 3GC-resistant strains had a MIC greater than 64 mg/L for cefotaxim and ceftazidime (85 and 67.5 %, respectively). This resistance was mostly due to the presence of an ESBL (*n* = 39, 85 %): 24 *K. pneumoniae*, 11 *E. coli*, 3 *Enterobacter spp.* and one *M. morganii.*

**Table 1:** Susceptibility of outpatients *Enterobacteriaceae* urinary isolates to various antimicrobial agents

	Number of isolates resistant to antibiotics (%)		
	*E. coli* (*n* =733)	Other Enterobacteriaceae (*n* =376)	All Enterobacteriaceae (*n* =1109)
Amoxicillin	42.1	88	57.6
Co-amoxiclav	18.6	32.7	23.4
Ticarcillin	38.9	75.8	51.4
Cefalothin	16.7	30	21.2
Cefoxitin	2.7	23.1	4.5
Cefotaxim	3.1	12.2	6.2
Ceftazidim	2.3	12.8	5.7
Aztreonam	1.9	11.7	7
Imipenem	−	6.7	2.3
Cotrimoxazole	18.6	16	17.8
Amikacin	0.4	0.5	0.5
Gentamicin	5.4	8.2	6.4
Nalidixic acid	19.7	21.3	20.2
Ciprofloxacin	13.8	19.4	15.6
Fosfomycin	1.6	17.9	7.1
Nitrofurantoin	1.2	32.6	13.7
Tetracyclin	25	32.7	27.6

Resistance to cotrimoxazole and ciprofloxacin was 17.8 and 15.6 %, respectively.

Resistance rates to fosfomycin and nitrofurantoin were low among *E. coli* strains (1.6 and 1.2 % respectively). These rates were higher for other *Enterobacteriaceae*: 17.9 % for fosfomycin and 32.6 % for nitrofurantoin.

Only a small proportion of *Enterobacteriaceae* strains were resistant to gentamicin (6.4 %) and amikacin (0.5 %) (Table 1). Only one strain (*Morganella morganii)* was resistant to imipenem.

Most 3GC-resistant strains had at least one co-acquired resistance: 85 % were resistant to ciprofloxacin, 67.5 % to cotrimoxazole and 52.5 % to gentamicin (Table 2).

**Table 2:** Characteristics of the ESBLE^a^

Species and ESBL type	Additionnal β lactamase			Acquired co-resistance^b^			
	non ESBL SHV	TEM1-like	OXA-1 like	SXT	GM	CIP	TE
*K. pneumoniae* (24)							
CTX M-15 (21)	16	11	11	14	11	21	11
CTX M-1 (2)	1	1	1	1	2	2	2
SHV-12 (1)	-	-	-	1	-	2	-
*E. coli* (11)							
CTX M-15 (5)	-	2	-	3	2	3	3
CTX M-1 (4)	-	3	-	4	2	1	3
CTX M-14 (1)	-	1	-	-	1	1	1
CTX M-27 (1)	-	1	1	1	-	1	1
*E. cloacae* (2)							
CTX M-15 (2)	-	2	2	2	1	2	1
*E. aerogenes*							
CTX M-1	-	-	-	-	-	-	-
*M. morganii*							
CTX M-15	-	-	-	1	1	1	1

^a^Number of strains is indicated in parentheses when >1

^b^ *SXT* cotrimoxazole, *GM* gentamicin, *CIP* ciprofloxacin, *TE* tetracyclin

### Determinants of antibiotic resistance in 3GC-R Enterobacteriaceae strains

The 39 ESBL producing strains are described in Table 2. Among them 38 expressed a CTX-M enzyme including 29 CTX-M15 and 7 CTX-M1. Additional β lactamases were found in most of strains (87.5 %).

### Risk factors for E. coli resistance to amoxicillin, third-generation cephalosporin and ciprofloxacin

Strains isolated from male patients had a significantly higher amoxicillin resistance rate than those isolated from female patients (*p* = 0.04). The ciprofloxacin resistance rate tended to increase with increasing age (*p* < 0.01) (Table 3).

**Table 3:** Analysis of independent risk factors for *E. coli* resistance to amoxicillin, trimethoprim/sulfamethoxazole and ciprofloxacin

Risk factor	Number	%	AMX				SXT				CIP			
			Crude OR [95 %]	*P* value	Ajusted OR [95 %]	*P* value	OR [95 %]	*P* value	Ajusted OR [95o%]	*P* value	OR [95 %]	*P* value	Ajusted OR [95 %]	*P* value
Sex - Male	124	19.9	1.5 [1.1–2.3]	0.04			1.4 [0.8–2.2]	0.21			1.6 [0.9–2.7]	0.09		
Age category														
less than 40 years	168	26.9	Ref				Ref				Ref			
40–59 years	141	23.7	0.9 [0.5–1.4]	0.55			1.1 [0.6–2.0]	0.81			2.7 [1.1–6.9]	0.03		
60–69 years	115	21.2	1.1 [0.7–1.8]	0.66			1.9 [1.1–3.4]	0.03			4.5 [1.9–10.9]	<0.01		
70 year and more	176	28.2	1.2 [0.7–1.8]	0.45			1.9 [1.1–3.3]	0.03			4.8 [2.1–11.3]	<0.01		
Presence of clinical signs	147	73.5	0.6 [0.4–1]	0.07	0.5 [0.2–0.9]	0.03	0.9 [0.3–3.1]	0.87			1.2 [0.6–2.6]	0.59		
Chronic illness	56	26.7	1.1 [0.7–1.9]	0.64			1.1 [0.3–3.7]	0.91			1.4 [0.7–2.7]	0.41		
Prior (3 months) UTI	52	24.8	1.8 [1–3.2]	0.04	2.3 [1.1–4.9]	0.02	3.1 [1–10.1]	0.05			5.4 [2.7–11]	<0.01	5.1 [2.5–10.3]	<0.01
Prior (12 months) hospitalization	32	15.2	2.2 [1.2–4.4]	0.02			5.1 [1.6–16.5]	<0.01			1.7 [0.8–3.7]	0.2		
Prior (6 months) urinary catheter	14	7	11.6 [2.7–50.6]	<0.01	8.8 [1.9–40.8]	<0.01	10.1 [2.5–40.4]	<0.01	11.1 [2.4–51.6]	<0.01	2.1 [0.7–5.7]	0.16		
Prior (6 months) antibiotic exposure	14	21.4	1.4 [0.8–2.4]	0.26			3.8 [1.1–12.9]	0.03	4 [0.9–18.9]	0.07	2.4 [1.2–4.9]	0.02		

Only significant adjusted OR are presented

*OR* odds ratio, *UTI* urinary tract infection

*Significant difference (*P* < 0.05)

Among 286 patients with an *Enterobacteriaceae*, 68 (23.7 %) declared to have received antibiotic treatment during the previous month and 72 (25.2 %) to have had a UTI during the three previous months. More *Enterobacteriaceae* strains isolated from patients who declared a UTI during the three previous months were resistant to amoxicillin (*p* = 0.02) and ciprofloxacin (*p* < 0.01) than those isolated from patients without a UTI during the three previous months. Moreover, urinary catheterization was associated with higher resistance rates for amoxicillin and cotrimoxazole (*p* < 0.01) (Table 3).

### Evolution of antibiotic resistance in E. coli from 2003

Resistance rates for amoxicillin, cotrimoxazole and amikacin have been stable during the last 10 years. On the contrary, we found a significant (*p* < 0.01) increase in the reported resistance to nalidixic acid, ciprofloxacin, cefalotin, and gentamycin (Table 4). The increase in the 3GC resistance rate was not significant as assessed by the Poisson regression test but was significant as evaluated by the Chi2 test. Until 2008, fewer than 1.0 % of *E. coli* strains were resistant to 3GC, but this proportion increased significantly between 2008 and 2009 (*p* < 0.01) and has been stable during the last 5 years (Table 4).

**Table 4:** Trends of resistance rates to various antimicrobial agents of *E. coli* between 2003 and 2014

Year	2003	2004	2005	2006	2007	2008	2009	2010	2011	2012	2013	2014	*P* value
n E.coli	198	300	338	437	384	399	606	763	854	766	1019	950	
Amoxicillin	43	46.6	43	42.3	43.2	44.1	39.7	37.7	45	44.2	41.4	42.9	NS
Cefalotin	8	6.3	7.8	7.3	5.5	6.2	15	14	16.6	17.6	17	20.4	<0.01*
Cefotaxime	0	0.7	0.3	0	0.9	0.2	1.8	2.5	2.7	3	2.6	2.3	<0.01**
Amikacin	0.5	0.0	0.0	0.5	0.6	0.2	0.2	1	0.7	0.3	0.3	0.3	NS
Gentamicin	1	3.3	4.4	2.3	2.6	4.2	3.6	3.9	5.6	5.2	3.4	4.6	<0.01*
Nalidixic acid	5.5	11	UD	UD	UD	13.4	15	13.1	13.7	17.2	16.2	20.8	<0.01*
Ciprofloxacin	4	5.3	6.8	8.5	9.6	8.5	11.4	8.2	10.9	9.9	9.9	12.3	<0.01*
Cotrimoxazole	18.7	23	21.6	22.4	23.2	23.3	20.9	19	20.5	19.4	18.6	18	NS

*UD* unavailable data, *NS* non significant

*P* values were calculated using the Poisson regression test (*) or the Chi2 test (**). Significant difference *P* < 0.05

## Discussion

As expected, *E. coli* was the most frequent strain isolated from urines in Guadeloupe (57.0 % of isolates), but nevertheless less frequent than in Europe and Canada (range from 61.0 to 87.5 %) [13–18]. *K. pneumoniae* was the second most frequent pathogen (15.5 %), even in pre-menopausal women, with a rate similar to those reported in Africa and Asia (ranging from 13.8 to 25.5 %) [6, 19–22]. *S. agalactiae* was the most frequent Gram-positive bacteria (4.3 %), whereas generally reported rates are less than 3 %. The differences in relative prevalence may be due to environmental and/or host genetic factors, or the virulence of the bacterial pathogens. Of note, the prevalence of diabetes is high (8.2 %) in Guadeloupe [23] and UTIs due to *K. pneumoniae* and *S. agalactiae* have been reported to be two to three times more frequent among patients with diabetes than those without [24].

As previously described, *S. saprophyticus* was isolated more frequently from women under 35 years of age than men or older women [25].

Overall, resistance rates were similar to those observed in developed countries including mainland France. Indeed, we found an amoxicillin-resistance rate of 42.1 % for *E. coli*, similar to the rate of 43.4 % reported for North America [26] and slightly lower than the rate of 48.3 % reported for Europe [27] . This contrasts with the reported situation in African and developing Asian countries where resistance rates may be as high as 80 % [6, 7, 28, 29].

Moreover, resistance to 3GC was low, and although this rate increased significantly between 2008 and 2009, it remains below 4 % for *E. coli* strains and 7 % for all *Enterobacteriaceae*. Guadeloupe should be considered to be a low prevalence ESBL-producing *Enterobacteriaceae* territory as are the European and North American countries [5, 15, 27, 30]. In our study, *bla*_CTX-M15_ was detected in most of the ESBL-producing strains (74.3 %), highlighting the shift during the 2000s from ESBL *bla*_TEM_/*bla*_SHV_ to ESBL group 1 *bla*_CTX-M_, and in particular to *bla*_CTX-M15_. This is in accordance with other epidemiological studies of ESBL-producing *Enterobacteriaceae* [31]. Most ESBL-producing strains (97.5 %) were resistant to other antibiotics in keeping with the ARESC and ECO-SENS II studies [5, 27]. Surprisingly, most ESBL-producing strains isolated in our study were *K. pneumoniae,* whereas in the community, *E. coli* strains with CTX-M enzymes usually predominate [32] because of the spread of clonal multidrug-resistant *E. coli* strains such as clone O25b-ST131 [33]. The prevalence of *K. pneumoniae* in our study can be explained not only by the high proportion of *K. pneumoniae* strains among uropathogens, but also by the spread of successful clones as described in Africa and Asia [34].

We did not isolate any carbapenemase-producing strains, although these strains have been observed in the community, even in low prevalence countries such as England [15] where their presence is often related to travel in Asia where these strains are endemic [35]. In Guadeloupe, *Enterobacteriaceae* carbapenemase-producing strains have just begun to emerge in hospital settings with the first description of an NDM-1-producing *K. pneumoniae* in 2014 [36], and more recently, of an OXA-48-producing *E. coli* [37].

Ciprofloxacin resistance remains moderate (15.6 %) compared to rates observed in Asia which can be as high as 75 % [19]. Nevertheless, we found that this rate has increased significantly during the last 10 years as described for other countries [6, 15, 26, 38]. Although we were not able to obtain retrospective data on fluoroquinolone consumption in Guadeloupe, increasing rates of ciprofloxacin-resistant strains suggest the inappropriate use of fluroquinolones in outpatients. Among the 48 patients reporting antibiotic consumption during the month prior to the infection, 15 had consumed a fluoroquinolone and among 24 patients that also reported a previous UTI, 12 received a fluoroquinolones whereas only one was given fosfomycin. Indeed, ciprofloxacin resistance in *E. coli* has been linked with ciprofloxacin consumption in the same month as the infection and the month before [39]. The rate of ciprofloxacin resistance tended to increase with age as has been described for mainland France and Canada [16, 40] Antimicrobial susceptibility testing of uropathogens is known to overestimate rates of resistance because antibiograms are performed mostly if empirical treatment fails, or if patients have underlying factors [41]. Nevertheless, the trends remain valid.

Consistent with previous reports [28, 42] we observed that prior UTI (likely to be associated with prior antibiotic treatment) was associated with higher amoxicillin and ciprofloxacin resistance rates. This suggests the abusive use of these antibiotics for the treatment of community acquired infections. Surprisingly, a previous antibiotic treatment was not associated with such a risk. Since the use of antibiotics for which the resistance rate exceeds 10–20 % is associated with an increased risk of treatment failure and the selection of resistant strains [43], ciprofloxacin should be administered only if compatible with the antimicrobial resistance pattern of the strain.

## Conclusions

Although a few patients may have been infected by hospital-acquired strains, this is the first description of susceptibility patterns of urinary bacterial strains isolated from outpatients in Guadeloupe.

The antibiotic resistance rates among urinary bacteria isolates from outpatients in Guadeloupe are similar to those of North America and Europe. Nevertheless, this study highlights the need for general practitioners in Guadeloupe to be better informed so that they are encouraged to prescribe fosfomycin-trometamol for the treatment of UTI to reduce the risk of increasing resistance rates to fluoroquinolones.

## Abbreviations

UTI, urinary tract infection; SPFIL, The French Language Infectious Disease Society; ESBL, Extended Spectrum beta lactamase; MIC, minimum inhibitory concentration; PCR, polymerase chain reaction; 3GC, third generation cephalosporin
